# Supplementary material for: A computational study of the Warburg effect identifies metabolic targets inhibiting cancer migration
Source: Mol Syst Biol. 2014 Aug 1;10(8):744. doi: 10.15252/msb.20134993 (PMC4299514; doi:10.15252/msb.20134993)
Supplement: Supplementary file 5 — Supplementary Information [file msb0010-0744-SD5.docx]

Supplementary Information

**A computational study of the Warburg effect identifies metabolic targets inhibiting cancer migration**

Keren Yizhak^1,7,*^, Sylvia E. Le Dévédec^2,*^, Vasiliki Maria Rogkoti^2^, Franziska Baenke^3^, Vincent C. de Boer^4^, Christian Frezza^5^, Almut Schulze^3^, Bob van de Water^2,¥^, Eytan Ruppin^1,6,7,¥^

^1^ The Blavatnik School of Computer Science, Tel-Aviv University, Tel-Aviv 69978, Israel

^2^ Division of Toxicology, Leiden Academic Centre for Drug Research, Leiden University, The Netherlands

^3^ Gene Expression Analysis Laboratory, Cancer Research UK, London Research Institute, London, UK

^4^ Laboratory Genetic Metabolic Diseases, Academic Medical Center, Amsterdam, The Netherlands

^5^ MRC Cancer Unit, University of Cambridge, Hutchison/MRC Research Centre, Box 197, Cambridge Biomedical Campus, Cambridge United Kingdom, CB2 0XZ

^6^ The Sackler School of Medicine, Tel-Aviv University, Tel-Aviv 69978, Israel

^7^ Corresponding authors: [kerenyiz@post.tau.ac.il](mailto:kerenyiz@post.tau.ac.il); [ruppin@post.tau.ac.il](mailto:ruppin@post.tau.ac.il)

^*^These authors contributed equally to this study

^¥^These authors contributed equally to this study

**Table of Contents**

1. The PRIME algorithm

- Supplementary Figure S1 – Biomass production as a function of the model's upper bound

1. Stoichiometric and flux capacity constraints successfully capture the coupling of high cell proliferation rate to lactate secretion across individual NCI-60 cancer models

- Supplementary Figure S2 – Lactate secretion as a function of glucose, glutamine and oxygen consumption rates

1. The different Warburg scores and their association with cell proliferation and cell migration

- Supplementary Figure S3 - Example of time-lapses of all 6 cell-lines
- Supplementary Table S1– Summary of association of the different indices with cell proliferation, cell migration and drug response

1. Analyzing individual fluxes and their relation to cell proliferation and cell migration

- Supplementary Figure S4 - Correlation of predicted fluxes with experimentally measured growth rate and migration speed

1. Predicting drug targets that revert the AFR and hence may inhibit cancer migration

- Supplementary Table S2 – List of predicted drug targets
- Supplementary Table S3**-** Correlation between the targets' gene expression and measured migration speed
- Supplementary Figure S5 – Western blot analysis of knockdown efficiency

1. ECAR and OCR levels following selected gene knockdown

- Supplementary Figure S6 – Defining optimal cell density for the measurement of OCR and ECAR using the Seahorse
- Supplementary Table S4 - Significance level of reduction in EOR following gene silencing in respect to control

1. References

**(1) The PRIME algorithm**

PRIME is given the following two inputs: (1) a set of *p* samples with gene expression levels and their corresponding growth rate measurements, and (2) a generic model (the human model, in our case). Next, the model reconstruction process is as follows:

1. Each reversible reaction is decomposed into its forward and backward direction and the maximal biomass production is evaluated. Next, the upper bound of all the reactions in the network is decreased simultaneously in steps of 0.1. In each step, the maximal biomass production is re-evaluated and the process proceeds as long as the reduction in bound doesn't decrease the maximal production found above. Finally, the upper bound of all reactions is set to the minimal upper bound allowed by this process. The goal of this step is to narrow down the solution space and reduce the effect of futile cycles.
2. Next, the correlation between the expression of each reaction in the network and the measured growth rates is evaluated. The expression of a given reaction is defined as the mean expression of its catalyzing enzymes. The significance threshold is set by an FDR analysis with α = 0.05.
3. The upper bound of each reaction demonstrating a significant correlation to the growth rate is modified in a manner that is linearly related to its expression value (e.g., *t* reactions). Specifically, the first step towards setting the upper bound of each reaction *i* in sample *j* is by defining the Exp-matrix, $E_{i,j}$:

$E_{i,j}= \frac{\rho_{i}}{|\rho_{i}|} \cdot\mathrm{GE}_{i,j}$(1)

In Equation (1), ${GE}_{i,j}$ represents the expression value of reaction *i* in sample *j*. Likewise, $\rho(i)$ represents the correlation coefficient of reaction *i* as found in step (2). The Exp-matrix, a $(t\times p)$ matrix, embeds the information on the direction and magnitude of change of the upper bound based on the expression data. Thus, for reactions whose expression is positively correlated with growth rate, the corresponding values in the matrix increase (become more positive) as the expression increases. Alternatively, for negatively correlated reactions, the corresponding values in the matrix decrease (become more negative) as the expression increases (due to the multiplication by $\frac{\rho_{i}}{|\rho_{i}|}$ which equals to -1 in this scenario).

We then apply Equation (2) to normalize the values of the Exp-matrix and adapt them to the actual upper bounds. In this normalization procedure each reaction *i* is normalized across its *p* samples such that the bound associated with the sample having the lowest (highest) expression value is assigned the minimal (maximal) value of the normalization range, respectively.

$UB_{i,j}= \left( \frac{E_{i,j}- min(E_{i})}{max{(E}_{i})- min(E_{i})}\cdot\left( maxNormVal-minNormVal \right) \right)+minNormVal$ (2)

$min{(E}_{i})$ and $max(E_{i}$) refer to the minimal and maximal value of reaction *i* across all *p* samples in the Exp-matrix, respectively. First, the minimal value of the normalization range (*minNormVal)* is set to be the minimal flux necessary for biomass production. This value is computed via Flux Variability Analysis ([Varma & Palsson, 1994](#_ENREF_3)) in the following manner: First, the set of essential reactions in the model is identified via Flux Balance Analysis. This set is composed of those reactions that their knock-out reduces growth by more than 90% of its maximal rate. Next, the minimal flux through each essential reaction is found via Flux Variability Analysis. As each of these reactions is necessary for biomass production, reducing the upper bound below their minimal flux value would result with a lethal phenotype. Finally, we set *minNormVal* to be the maximal value among these values. To define the maximal value of the normalization range (*maxNormVal*) we examine the change in biomass production as a function of the model's upper bounds. The following steps are applied:

1. First, we define the set of reactions in the model that are significantly correlated to the growth rate (as described in step (2) above).
2. Next, we examine how the biomass production is changed as a function of the model's upper bound. This is done by changing the upper bounds of the reactions found in (a) in steps of 0.1, and in each step re-evaluating the biomass production. Supplementary Figure S1 demonstrates the relation between upper bound and biomass production.
3. Lastly, *maxNormVal* is defined as the maximal value beyond which the change in biomass production decrease (i.e., the slope in Supplementary Figure S1 becomes smaller).

**
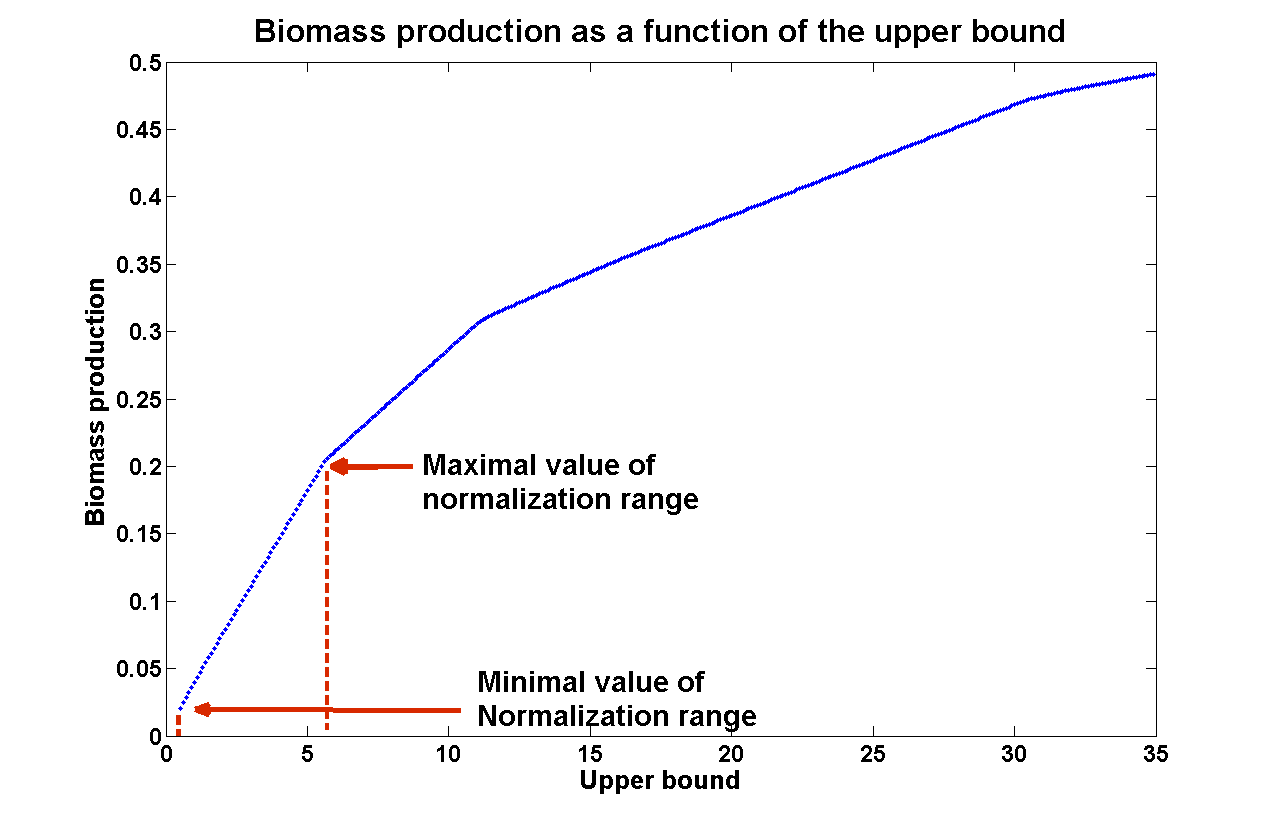
**

**Supplementary Figure S1:** Biomass production as a function of the model's upper bound.

**(2) Stoichiometric and flux capacity constraints successfully capture the coupling of high cell proliferation rate to lactate secretion across individual NCI-60 cancer models**

To assess the extent of lactate secretion and its breadth we calculated its minimal secretion rate in each of our NCI-60 cell-line models under more than 10000 simulated different growth media conditions. These conditions included different uptake rates of glucose (0 to 20 mmol/gDW/h), glutamine (0 to 10 mmol/gDW/h) and oxygen (0 to 10 mmol/gDW/h), as well as different demands for minimal biomass production rate (0% to 100%). As depicted in Supplementary Figure S2A, high levels of glucose uptake result with lactate secretion even in the presence of oxygen, across a wide range of these alternative media conditions. Further, we find that lactate secretion is by and large a byproduct of glucose rather than of glutamine metabolism ([DeBerardinis et al, 2008](#_ENREF_2)) (Supplementary Figure S2B).


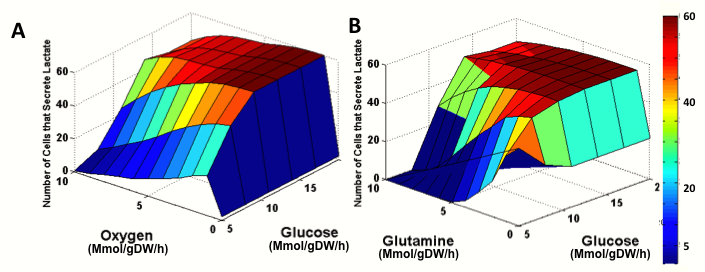


**Supplementary Figure S2:** The number of NCI-60 cell-lines that must secrete lactate when enforcing biomass production, as a function of glucose, glutamine and oxygen consumption. **(A)** High levels glucose uptake result in lactate secretion to various degrees even in the presence of oxygen. **(B)** Glucose and not glutamine is a key determinant of lactate secretion, as even at high glutamine uptake rates, lactate secretion rate can equal zero. Results are presented at the model's flux units, mmol/gDW/h.

**(3) The different Warburg scores and their association with cell proliferation and cell migration**


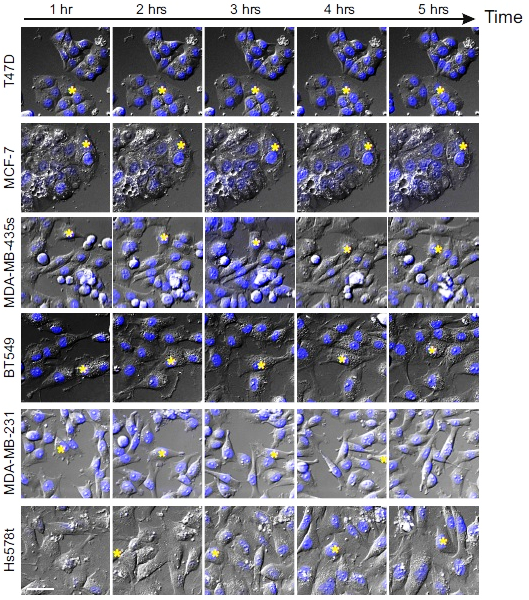


**Supplementary Figure S3:** Example of time-lapses of all 6 breast cancer cell-lines. Snapshots of 5 hours of imaging period for the different cell-lines. For cell tracking, the nuclei were live stained with Hoechst. Cells were then followed for 12 hours using epifluorescent and DIC imaging; the yellow star is one typical cell followed over time; scale bar is 10 𝜇m.

Supplementary Table S1 – Summary of association of the different indices with cell proliferation, cell migration and drug response

| **Index** | **Correlation to growth** | | **Correlation to migration** | | **Partial correlation to migration** | | **Percentage of compounds showing a significant difference between low/high Warburg level** |
| --- | --- | --- | --- | --- | --- | --- | --- |
|  | Rho | P-value | Rho | P-value | Rho | P-value |  |
| **AFR** | -0.55 | 4.53e-6 | 0.88 | 0.03 | 0.96 | 7.6e-3 | 30% |
| **EOR** | -0.64 | 3.38e-8 | 0.77 | 0.1 | 0.71 | 0.17 | 19% |
| **BEC** | -0.43 | 4.76e-4 | 1 | 0.002 | 1 | <1e-4 | 7.5% |

**(4) Analyzing individual fluxes and their relation to cell proliferation and cell migration**

The association between increased AFR and cellular migration may seem surprising at first, as increased glycolysis was shown to support cell growth by diverting carbon from glucose toward the synthesis of molecular building blocks. ([Benjamin et al, 2012](#_ENREF_1)). Analyzing the predicted flux rates across the models sheds further light on this seemingly paradoxical finding. While the fluxes carried by the glycolysis are negatively (positively) correlated with growth (migration), respectively, the fluxes carried by the biosynthetic pathways that branch from glycolysis indeed exhibit a positive correlation to growth and a negative one to migration (Supplementary Figure S4). Our findings suggest that re-routing glycolysis-related fluxes play a key role in shaping the dichotomy between cell proliferation and cell migration.


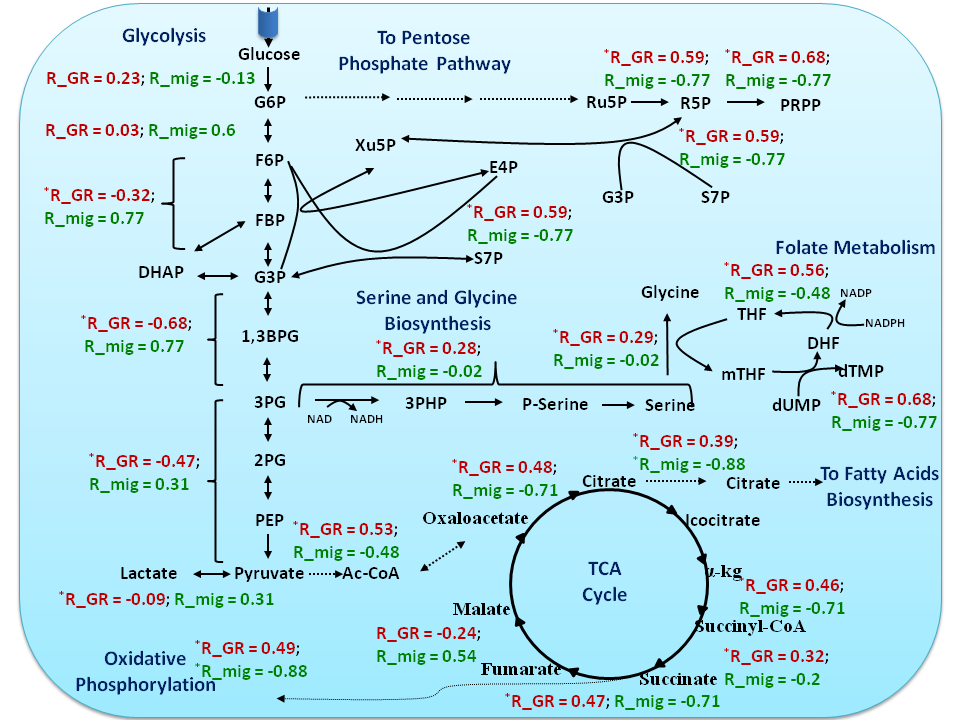


**Supplementary Figure S4:** Correlation of predicted fluxes with experimentally measured growth rate (R_GR, red) and migration (R_mig, green) across central metabolic pathways: glycolysis, serine and glycine biosynthesis, pentose phosphate pathway, TCA cycle and OXPHOS. The glycolysis fluxes exhibit a positive/negative correlation to growth rate and migration, respectively, while an opposite trend is observed in the pentose phosphate pathway, serine and glycine biosynthesis, folate metabolism, the TCA cycle and OXPHOS. Significant correlations are marked with an asterisk. Of note, due to the small sample size of the migration dataset the correlations are mostly insignificant when taken individually, but are still presented to make their overall aggregated effect apparent. It should be noted that growth rate comparison was done across all 60 cell-lines and the migration comparison for only the 6 available cell-lines.

**(5) Predicting drug targets that revert the AFR and hence may inhibit cancer migration**

Supplementary Table S2 – List of predicted drug targets

| **Metabolic Pathway** | **Enzyme** | **Reaction Description** |
| --- | --- | --- |
| Methionine Metabolism | AHCY | ahcys[c] + h2o[c] <=> adn[c] + hcys_DASH_L[c] |
| Methionine Metabolism | MAT1A/MAT2A | atp[c] + h2o[c] + met_DASH_L[c] => amet[c] + pi[c] + ppi[c] |
| Glycolysis/Gluconeogenesis | GAPDH | g3p[c] + nad[c] + pi[c] <=> 13dpg[c] + h[c] + nadh[c] |
| Glycolysis/Gluconeogenesis | PKM2 | adp[c] + h[c] + pep[c] => atp[c] + pyr[c] |
| Glycolysis/Gluconeogenesis | ENO1/ENO2/ENO3 | 2pg[c] <=> h2o[c] + pep[c] |
| Glycolysis/Gluconeogenesis | TPI1 | dhap[c] <=> g3p[c] |
| Glycolysis/Gluconeogenesis | PGAM1/PGAM2 | 2pg[c] <=> 3pg[c] |
| Glycolysis/Gluconeogenesis | PGK1/PGK2 | 3pg[c] + atp[c] <=> 13dpg[c] + adp[c] |
| Glycolysis/Gluconeogenesis | HK2 | atp[c] + glc_DASH_D[c] => adp[c] + g6p[c] + h[c] |
| Glycine, Serine, and Threonine Metabolism | PSPH | h2o[c] + pser_DASH_L[c] => pi[c] + ser_DASH_L[c] |
| Glycine, Serine, and Threonine Metabolism | PSAT1 | 3php[c] + glu_DASH_L[c] => akg[c] + pser_DASH_L[c] |
| Glycine, Serine, and Threonine Metabolism | PHGDH | 3pg[c] + nad[c] => 3php[c] + h[c] + nadh[c] |

Supplementary Table S3 **-** Correlation between the targets' gene expression and measured migration speed across the NCI-60 cell-lines

| **Gene Name** | **Spearman Rank Correlation (Rho)** | **P-value** |
| --- | --- | --- |
| AHCY | -0.77 | 0.10 |
| MAT1A | -0.25 | 0.65 |
| MAT2A | -0.65 | 0.17 |
| GAPDH | 0.08 | 0.91 |
| PKM | 0.08 | 0.91 |
| ENO1 | 0.77 | 0.10 |
| ENO2 | 0.54 | 0.29 |
| ENO3 | -0.31 | 0.56 |
| TPI1 | -0.08 | 0.91 |
| PGAM1 | -0.37 | 0.49 |
| PGAM2 | 0.14 | 0.8 |
| PGK1 | 0.37 | 0.49 |
| PGK2 | -0.08 | 0.91 |
| HK2 | 0.02 | 1 |
| PSPH | 0.77 | 0.10 |
| PSAT1 | 0.94 | 0.01 |
| PHGDH | 0.42 | 0.41 |


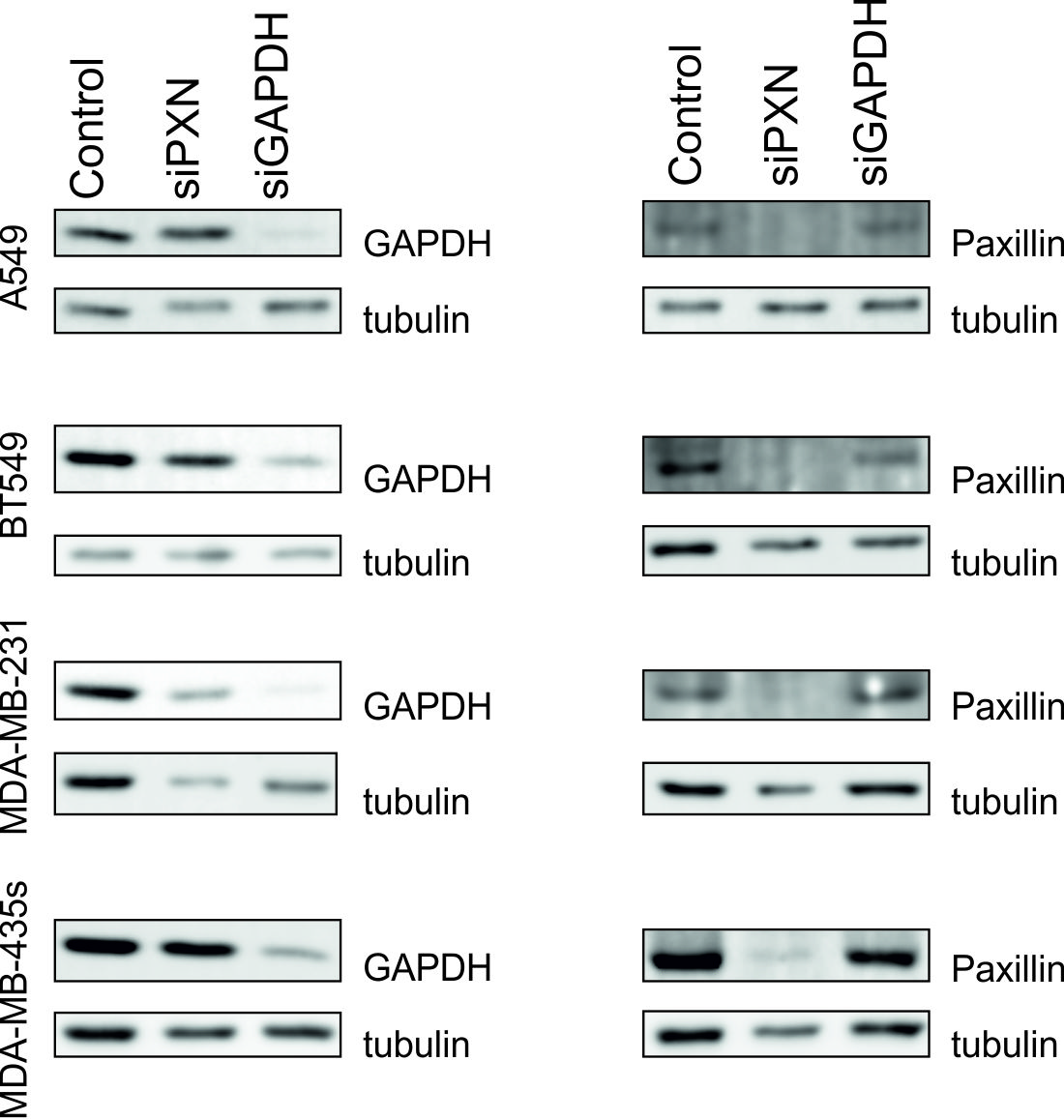


**Supplementary Figure S5:** Western blots of paxillin and GAPDH after their respective knockdown in all four cell lines used for the RNA interference screen. Cells were harvested 72 hours after siRNA transfection and analysed for the knockdown efficiency of both paxillin and GAPDH. This analysis confirms that our knockdown protocol works efficiently at least for those genes.

**ECAR and OCR levels following selected gene silencing**


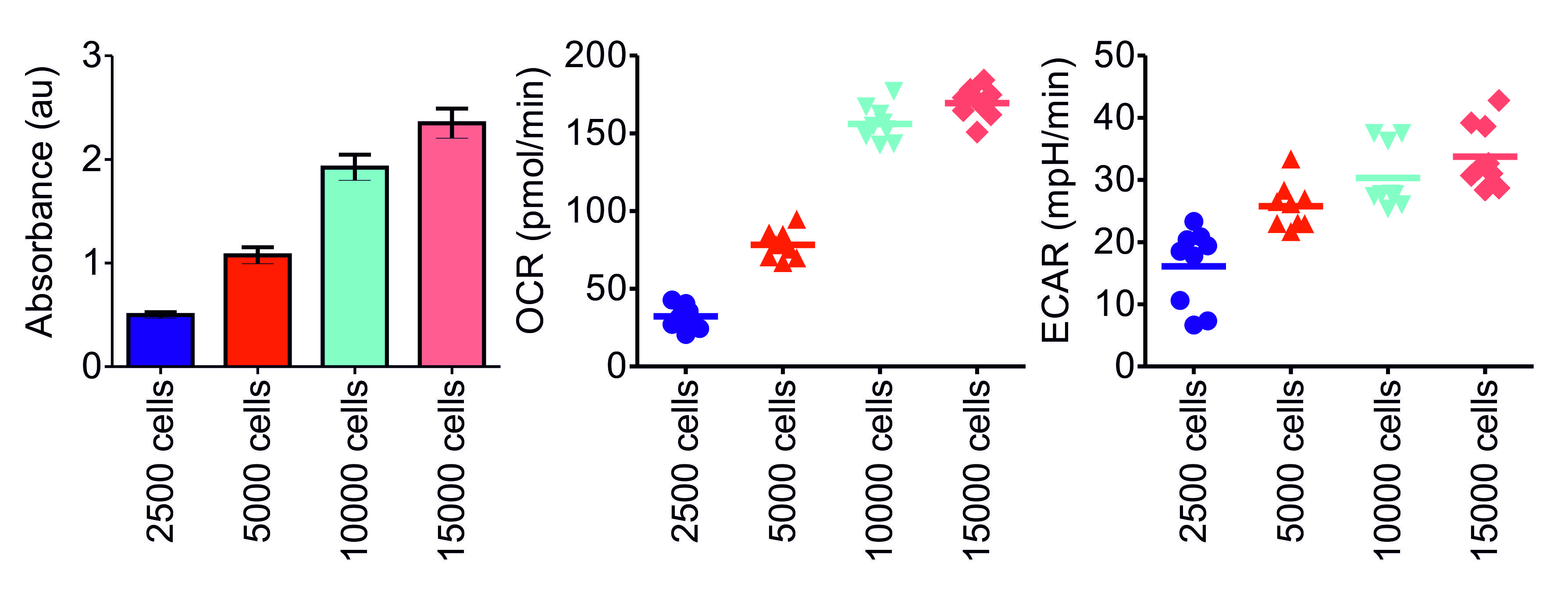


**Supplementary Figure S6:** Determining the most optimal density of MDA-MB-231 cells for OCR and ECAR measurements. The number of cells was verified in each well of the Seahorse plate with the SRB assay. There is clear correlation between the plated densities and the measured protein content. Based on the OCR and ECAR detected levels with the Seahorse, we decided to use a cell density between 5000 and 10 000 cells/well for the knockdown experiment.

Supplementary Table S4 **–** Significance level of reduction in EOR following gene silencing in respect to control

| **Gene Name** | **Two-sided T-test P-value** |
| --- | --- |
| HK2 | 1.4e-14 |
| PGAM1 | 3.09e-11 |
| PGK2 | 1.04e-10 |
| GAPDH | 5.9e-5 |
| PSPH | 7e-3 |
| AHCY | 0.3 |
| PHGDH | 0.04 |

**References**

Benjamin DI, Cravatt BF, Nomura DK (2012) Global Profiling Strategies for Mapping Dysregulated Metabolic Pathways in Cancer. *Cell Metabolism* **16:** 565-577

DeBerardinis RJ, Sayed N, Ditsworth D, Thompson CB (2008) Brick by brick: metabolism and tumor cell growth. *Current Opinion in Genetics & Development* **18:** 54-61

Varma A, Palsson BO (1994) Metabolic flux balancing: Basic concepts, scientific and practical use. *Bio Technology* **12:** 994-998
